# TNF+ regulatory T cells regulate the stemness of gastric cancer cells through the IL13/STAT3 pathway

**DOI:** 10.3389/fonc.2023.1162938

**Published:** 2023-07-18

**Authors:** Rou Zhao, Guanjie Cao, Baogui Zhang, Li Wei, Xiaobei Zhang, Meng Jin, Baoyu He, Bin Zhang, Zhun He, Qingli Bie

**Affiliations:** ^1^ Department of Laboratory Medicine, Affiliated Hospital of Jining Medical University, Jining Medical University, Jining, Shandong, China; ^2^ Department of Radiology, Affiliated Hospital of Jining Medical University, Jining Medical University, Jining, Shandong, China; ^3^ Colorectal Ward, Department of Gastrointestinal Surgery, Affiliated Hospital of Jining Medical University, Jining Medical University, Jining, Shandong, China; ^4^ Department of Pathology, Affiliated Hospital of Jining Medical University, Jining Medical University, Jining, Shandong, China; ^5^ Medical Research Center, Affiliated Hospital of Jining Medical University, Jining Medical University, Jining, Shandong, China; ^6^ Hernia and Abdominal Wall Surgery, Affiliated Hospital of Jining Medical University, Jining, Shandong, China; ^7^ Department of Thoracic Surgery, Affiliated Hospital of Jining Medical University, Jining Medical University, Jining, Shandong, China

**Keywords:** TNF, Tregs, gastric cancer, cancer stem cell, IL-13

## Abstract

Regulatory T cells (Tregs) are an important component of the tumor microenvironment; however, the interaction between Tregs and gastric cancer cells is not completely understood. Recent studies have shown that Tregs participate in cancer cell stemness maintenance. In this study, we performed single-cell RNA sequencing of gastric cancer and adjacent tissues and found that Tregs with high TNF expression were recruited to gastric cancer tissues and were significantly correlated with patient survival. TNF+ Tregs significantly contribute to tumor growth and progression. Our studies have further demonstrated that TNF+ Tregs promote the stemness of gastric cancer cells through the IL13/STAT3 pathway. Therefore, blocking the interaction between TNF+ Tregs and gastric cancer cells may be a new approach in the treatment of gastric cancer.

## 1 Introduction

There are more than 1 million new cases of gastric cancer every year (1). Although the treatment of gastric cancer has made great progress in recent decades, due to the limitations of various treatment methods and the characteristics of tumor recurrence and metastasis, the current treatment outcomes for gastric cancer are still unsatisfactory (2). The occurrence and development of gastric cancer is a complex process involving multiple factors and multiple steps, among which the acquisition of stem-like properties of tumor cells is closely related to the occurrence and development of gastric cancer (3).

Regulatory T cells (Tregs) are a type of lymphocyte that negatively regulates the immune response. They usually play critical roles in maintaining autoimmune tolerance and avoiding excessive immune response damage, but they also participate in tumor immune escape (4). The forkhead family transcription factor P3 (Foxp3) is essential in the development and differentiation of Tregs. As the main marker of Tregs, Foxp3 strongly regulates the function and plasticity of Tregs (5). In addition, CD25 can be used as a marker for Tregs (6).

Tregs in the tumor microenvironment can inhibit the antitumor response, thus promoting tumor growth (7). Studies have revealed that immune cells in the tumor microenvironment can directly or indirectly enhance the stemness of gastric cancer cells (8). Tumor-associated macrophages (TAMs) modulate the tumorigenicity and drug resistance of cancer stem cells (CSCs) through lactoglobulin epidermal growth factor VIII (MFG-E8) (9). Tumor-induced STAT3 activation in MDSCs enhances stemness and mesenchymal properties in pancreatic cancer (10). Tregs can inhibit monocytes/macrophages, dendritic cells, B cells and other immunocompetent cells through cytokine secretion and cytotoxicity (11). More importantly, Tregs have been proven to promote the stemness of tumor cells, including those in leukemia (12), breast cancer (13) and glioma (14). However, the mechanisms whereby Tregs alter the properties of gastric cancer cells are still obscure.

In this study, we performed single-cell sequencing of gastric cancer and adjacent tissues and found a subpopulation of TNF+ Tregs in GC tumor microenvironment. We confirmed the role of TNF+ Tregs in promoting the stemness of gastric cancer cells and explored the mechanism by which TNF+ Tregs affect gastric cancer cells. We found that TNF+ Tregs released a high level of the anti-inflammatory cytokine IL13, which promotes the malignant biological function of gastric cancer cells by activating STAT3. This study shows that the development of communication strategies between Tregs and gastric cancer cells is crucial to preventing the progression of gastric cancer.

## 2 Result

### 2.1 Analysis of Tregs in the GC tumor microenvironment

**Figure 1:**
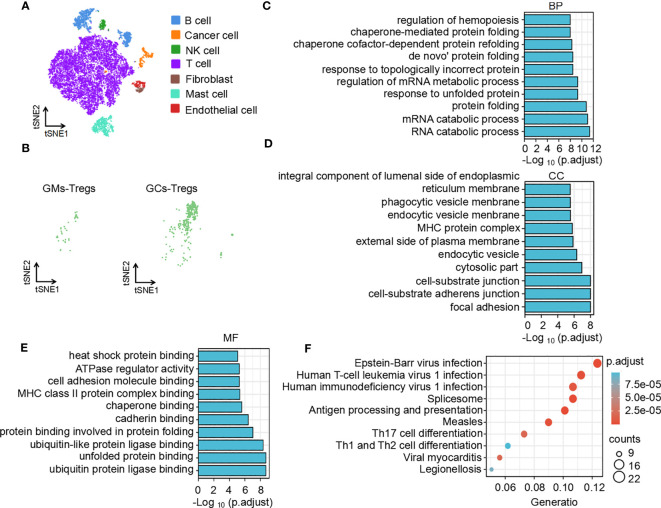
Function and pathway enrichment analysis of DEGs in Tregs in the GC tumor microenvironment. **(A)** The tSNE maps in tissue samples. **(B)** Distribution of Tregs in tumors and adjacent normal tissues. Top 10 biological processes **(C)**, cellular components **(D)**, molecular functions **(E)** and signaling pathways **(F)** of upregulated differentially expressed genes in Tregs in the GC tumor microenvironment.

To understand the difference in the immune microenvironment between tumor tissue and adjacent tissue of gastric cancer, we performed single-cell dissociation, sorting, transcriptome library construction and sequencing of gastric cancer (GC) tissues and gastric mucosa (GM) tissues. After integrating the data, filtering low-quality cells, removing multiple cells, and correcting batch effects, the transcriptome expression matrix was finally obtained. Then, tSNE dimensionality reduction and visual analysis were performed on the expression matrix. According to the canonical cell labeling listed in **Table 1** (the violin plot with marker gene expression is shown in **Supplementary Figure 1**), the cells in GC and GM tissues were classified as B cells, cancer cells, NK cells, T cells, fibroblasts, mast cells and endothelial cells (**Figure 1A**).

**Table 1:** Cell markers for cell population identification.

Cell Type	Markers
B cells	CD79A, CD19
T cells	CD3D
NK cells	CD16, CD56
Mast cells	CPA3
Fibroblasts	CD90, COL1A1, COL3A1
Endothelial cells	CD34, CD31,VWF
Cancer cells	CA9, CD24
Tregs	FOXP3, CD25

Based on the expression of cell markers (**Table 1**), Tregs were further identified (**Figure 1B**). Then, we identified the differentially expressed genes in Tregs; the heatmap in **Supplementary Figure 2A** shows the upregulated genes in Tregs between GCs and GM tissues. GO and KEGG analyses were used to annotate the functions of the significantly upregulated genes in Tregs of the gastric cancer microenvironment. GO analysis showed that the upregulated genes were mainly enriched in the regulation of mRNA processes, protein binding or folding and cell junctions, and adhesion (**Figures 1C–E**). KEGG pathway analysis showed that the upregulated genes were implicated in regulating of Th17 and Th1 and Th2 cell differentiation (**Figure 1F**). Taken together, the single-cell sequencing analysis results indicate that Tregs may play an important role in immune regulation in the GC immune microenvironment.

### 2.2 More TNF+ Tregs infiltrate in GC tumor tissues

**Figure 2:**
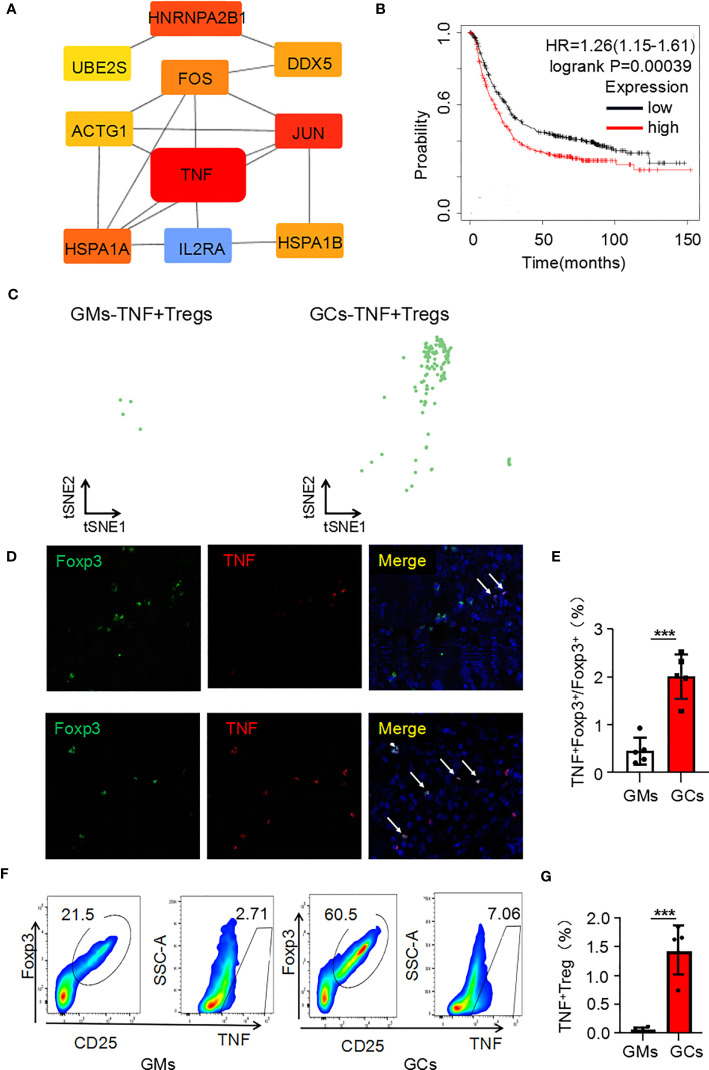
*Tnf* is highly expressed in Tregs of the GC microenvironment. **(A)** Hub genes of upregulated genes in Tregs in the GC tumor microenvironment. **(B)** Kaplan–Meier survival curves comparing high and low expression of *tnf* in gastric cancer. The red curve represents patients with high expression of *tnf*. **(C)** Distribution of TNF+ Tregs in tumors and adjacent normal tissues. **(D)** Representative photomicrographs of multiplex immunofluorescence. DAPI: nuclear staining (blue signal) TNF: red signal; FOXP3: green signal. **(E)** Analysis of the ratio of TNF+FOXP3+ cells to FOXP3+ cells in each high-power visual field for the experiment in panel C (n=5). **(F)** TNF+ Treg cells (flow analysis gating strategy: CD45+CD25+FOXP3+ cells were divided into Tregs, and then TNF was detected) in GC tissues and adjacent tissues were detected by flow cytometry. **(G)** Statistical analysis of the proportion of CD45+CD25+FOXP3+TNF+ cells according to **(E)** (n=4). ***p < 0.001.

It is the most essential work to identify hub genes from common DEGs to explore the key functions of cells (15). CytoHubba was used to trace the hub genes among the upregulated genes of Tregs in the GC tumor microenvironment. The hub genes were sorted by their BottleNeck value, and the differentially expressed genes with high BottleNeck values tended to correspond to highly central proteins that connect several complexes (16). The top ten identified hub genes were *tnf*, *jun*, *hnrnpa2/b1*, *hspa1a*, *fos*, *hspa1b*, *ddx5*, *actg1*, *ube2s*, and *il2ra* and *tnf* interacts with most other genes (**Figure 2A**). Among the 10 candidate genes, *tnf* had the highest expression level in TNF+ Tregs (**Supplementary Figure 2B**). We speculate that *tnf* may be the main key gene of Tregs in GCs. Further, KMplotter dataset and TCGA database analysis revealed that *tnf* was highly expressed in GCs and was significantly related to the low survival rate of gastric cancer patients (**Supplementary Figure 2C**; **Figure 2B**). The survival rate of cancer patients is related to the number and activation status of immune cells in the tumor (17). We calculated the quantity of immune cells *via* TIMER to explore whether the expression of *tnf* were related to immune cell infiltrated in GC. The results in **Supplementary Figure 2D** indicated that *tnf* expression was significant positive correlation with the infiltration of Tregs (R = 0.322, p = 1.281E–10). **Supplementary Figure 2E** shows that the infiltration of Tregs in GCs with high *tnf* expression was significantly higher than that in the low *tnf* expression group. These findings suggest that *tnf* might be related to the infiltration of Tregs in GCs. We further explored the relationship between *tnf* expression and Treg infiltration in GCs, and surprisingly, we found that the number of TNF+ Tregs in the tumor was 137, which was significantly higher than that in the adjacent normal tissues (4 TNF+ Tregs) (**Figure 2C**); this indicates the existence of a Treg subpopulation expressing *tnf.* To verify the above speculation, we performed multicolor immunofluorescence and found that the number of infiltrated TNF+ Tregs in GC tissues was significantly higher than that in GM tissues (**Figures 2D, E**). The results were further verified by flow cytometry (**Figures 2F, G**). The above results revealed that GC tissues have higher infiltration of TNF+ Treg subpopulations than GM tissues.

### 2.3 TNF+ Tregs promote malignant biological behaviors of GC cells

**Figure 3:**
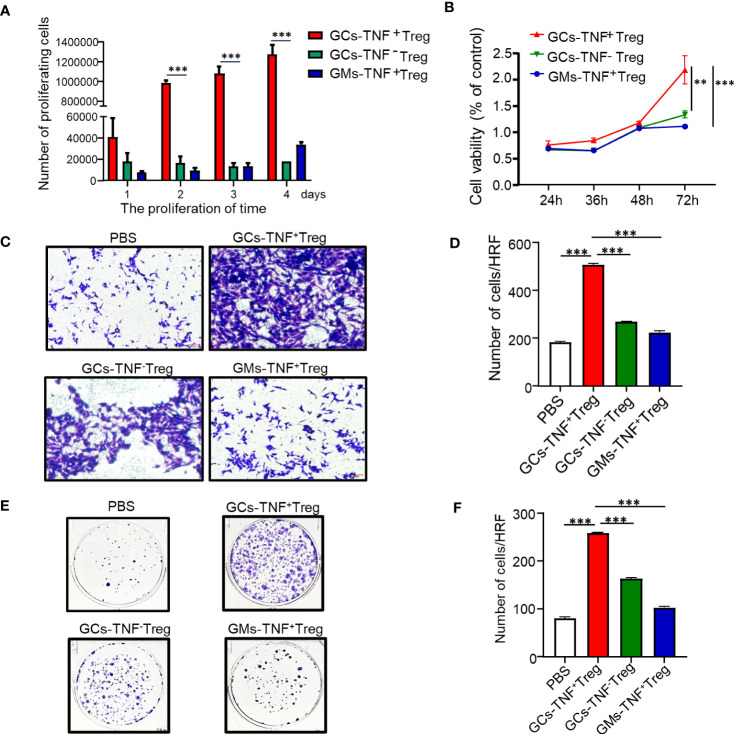
Effects of GC-TNF+ Treg cells on the proliferation and migration of gastric cancer cells. TNF+ Tregs and TNF- Tregs of gastric cancer and adjacent tissues were separated by flow cytometry and cultured in medium. **(A)** The number of Tregs was counted at 1, 2, 3 and 4 days (n=3). The supernatants of TNF+ Tregs and TNF-Tregs from gastric cancer and adjacent tissues were collected to stimulate HGC-27 cells for **(B)** cell viability (n=3), **(C, D)** cell migration (n=3), and **(E, F)** colony formation assays (n=3). ***p < 0.001, **p < 0.01.

To further explore the biological function of TNF+ Tregs, we first obtained TNF+ Tregs from gastric cancer tumor tissues of patients by flow cytometry sorting. As shown in **Figure 3A**, the proliferative capacity of TNF+ Tregs in GCs was obviously higher than that of TNF- Tregs in GC tissues and TNF+ Tregs in GM tissues. Cell culture supernatants of TNF+ Tregs and TNF-Tregs in GC tissues and TNF+ Tregs GM tissues were also collected, and the culture supernatant of TNF+ Tregs in GC tissues significantly promoted the proliferation, migration and colony formation of gastric cancer cells (**Figures 3B–F**). The above results indicate that TNF+ Tregs have important biological functions in gastric cancer and can promote the development of gastric cancer.

**Figure 4:**
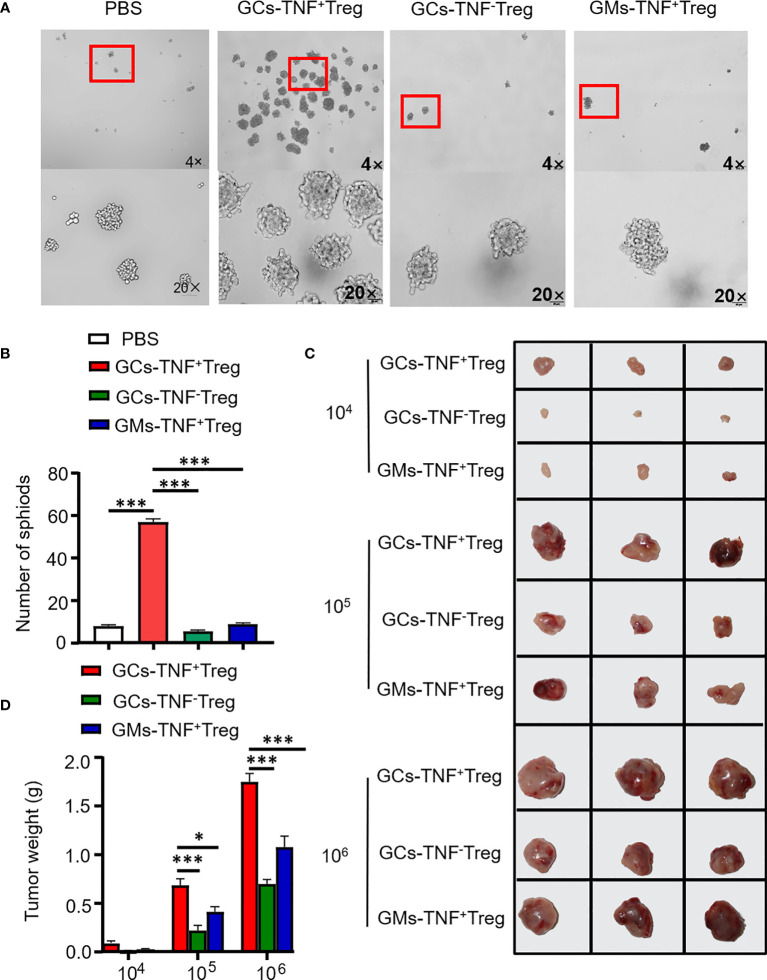
TNF+ Tregs promote the stemness of gastric cancer cells. Flow sorting of TNF+ Tregs and TNF- Tregs from gastric cancer and adjacent tissues was performed, and the cells were cultured in medium; the supernatant was collected to stimulate AGS and HGC-27 cells. **(A)** AGS cells were cultured in serum-free medium for 10 days (n=3). **(B)** Statistical analysis was performed on the number of spheroids (diameter > 50 μm). **(C)** HGC-27 cells were diluted and subcutaneously injected into mice with severe immunodeficiency (n=3). **(D)** The tumor volume was monitored in the indicated groups. ***p < 0.001, *p < 0.05.

Recent studies have demonstrated that Tregs endow cancer cells with stem cell-like properties and are linked with cancer stemness (17). In addition, TNF has been confirmed to confer cancer cell stemness properties (18–20). Therefore, we examined the effect of supernatant from TNF+ Tregs in GCs on AGS cell stemness. Compared with the supernatant of TNF- Tregs in GCs and TNF+ Tregs in GM tissues, the supernatant of TNF+ Tregs in GCs significantly promoted the self-renewal ability of AGS cells (**Figures 4A, B**). We established a xenograft tumor model with HGC-27 cells treated with TNF+ Treg/TNF- Treg supernatant in NVSG mice by subcutaneous injection. Mice were housed under exactly the same conditions. After 28 days, the mice were killed, and the tumors were removed, photographed, and analyzed. As shown in **Figures 4C, D**, HGC-27 cells stimulated by the supernatant of TNF+ Tregs in GCs had significantly increased tumorigenicity and tumor weight. The above results further confirmed the promotion effects of TNF+ Treg infiltrated in GC on the malignant biological behaviors of GC cell.

### 2.4 TNF+ Tregs regulate the stemness of gastric cancer cells through the IL-13-STAT3 pathway

**Figure 5:**
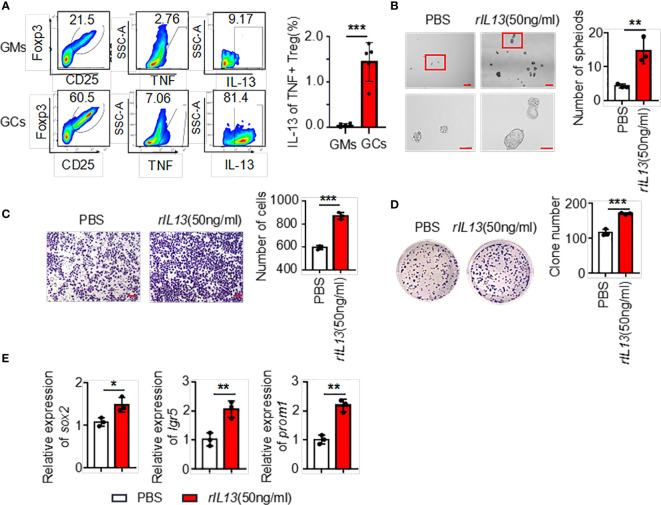
**(A)** The ratio of IL13 secreted by TNF+ Tregs (flow analysis gating strategy: CD45+CD25+FOXP3+ cells were divided into Tregs, then gate TNF+ Tregs, and finally gate IL13) in tumor tissues and adjacent tissues of gastric cancer was detected by flow cytometry. Statistical analysis of the original results (n=4). **(B)** Representative images of AGS cells cultured in serum-free medium for 7 days and stimulated with rIL13 or PBS. Statistical analysis of the spheroid number (diameter > 50 μm). PBS- or rIL13-stimulated AGS cells were prepared for **(C)** cell migration assay (n=3) and **(D)** colony formation assay (n=3). **(E)** qRT‒PCR was used to detect the expression of sox2, lgr5 and prom1 in AGS cells treated with PBS or rIL13 (n=3). ***p < 0.001, **p < 0.01, *p < 0.05.

**Figure 6:**
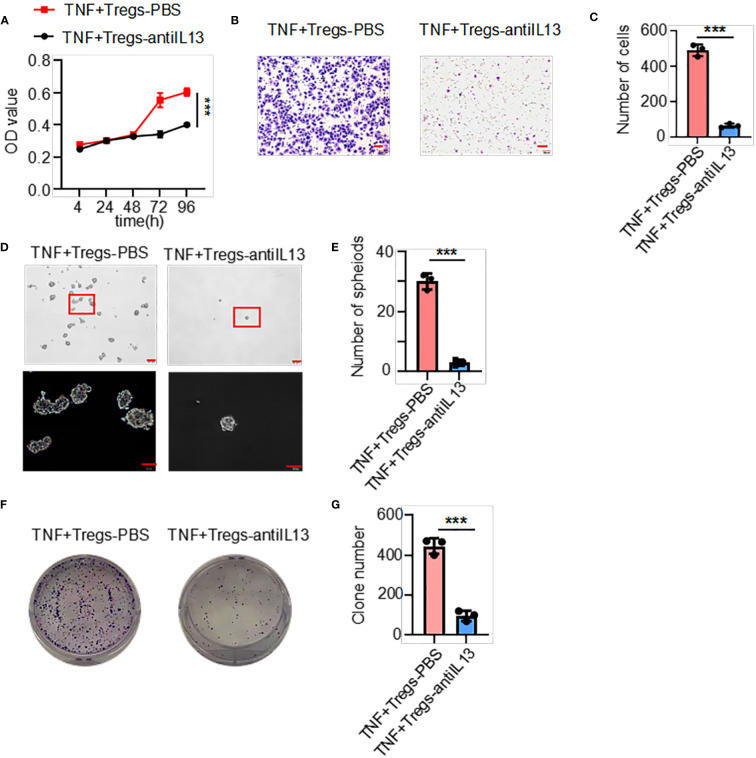
IL13 in the supernatant of TNF+ Tregs increased the malignant of gastric cancer cells. Gastric cancer cells were stimulated with supernatants of TNF+ Tregs from tumors and treated with anti-IL13 (20 μg/ml) to detect **(A)** cell viability (n=3), **(B, C)** cell migration (n=3), and **(D, E)** spheroid formation (n=3) and colony formation **(F, G)** (n=3). ***p < 0.001.

The above results showed that the supernatants of TNF+ Tregs promoted the malignant biological function of GC; therefore, we speculate that the components in the supernatant played an essential role. We collected TNF+ Tregs and TNF- Tregs sorted from tumor tissues and GM tissues by flow cytometry sorting. IL13 is involved in the maintenance of cellular stemness, and our previous study demonstrated that IL13 promotes tumor stemness (21). In addition, high IL-13 levels were closely associated with a high level of Tregs in the tumor microenvironment (22). As shown in **Figure 5A**, IL-13 expression in TNF+ Tregs of GC tissues was significantly higher than that in TNF+ Tregs of GM tissues, and IL13 levels in the supernatant of TNF+ Tregs in GC tissues were significantly higher than those in the supernatant of TNF- Tregs in GCs and TNF+ Tregs in GM tissues (**Supplementary Figure 3**). Therefore, IL-13 may be an important factor by which TNF+ Tregs regulate GC malignant biological function. Next, we studied whether IL13 could affect GC cells. We treated the cells with recombinant IL-13 (rIL-13). As shown in **Figures 5B–D**, rIL-13 significantly promoted the self-renewal, migration and colony formation of gastric cancer cells. Consistently, rIL13 promoted the expression of stemness-related genes, including *sox2*, *lgr5* and *prom1 (* **Figure 5E**). Moreover, to further explore the effects of IL13 in the supernatant of TNF+Tregs on GC cells, we treated cells with a neutralizing antibody against IL13 and showed that neutralizing IL13 significantly attenuated the effects of the supernatant from TNF+ Tregs on the proliferation, migration, sphere formation and colony formation of gastric cancer cells (**Figures 6A–G**). This suggests that the effect of TNF+ Tregs on gastric cancer cells is dependent on IL13.

**Figure 7:**
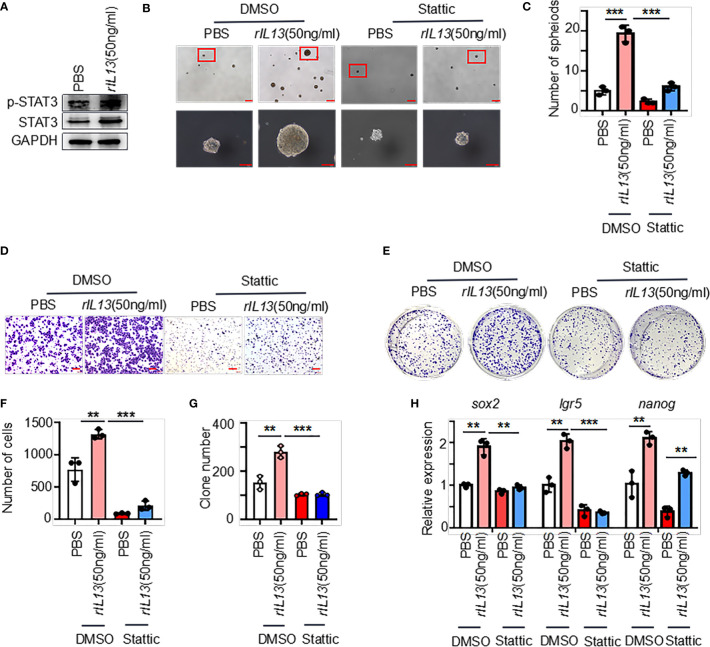
IL-13 plays a role in promoting the malignancy of gastric cancer cells through STAT3. **(A)** The protein levels of p-STAT3 and STAT3 were measured in AGS cells treated with PBS or rIL13. **(B)** Representative images of AGS cells cultured in serum-free medium for 7 days and treated with PBS/rIL13 and DMSO/stattic (n=3). **(C)** Statistical analysis of the spheroid number (diameter > 50 μm). PBS/rIL13- and DMSO/stattic-stimulated AGS cells for **(D)** cell migration assay (n=3) and **(E)** colony formation assay (n=3). Statistical analysis of Figure 7D **(F)** and 7E **(G)**. **(H)** qRT−PCR was used to detect the expression of *sox2*, *lgr5* and *nanog* in AGS cells treated with PBS/rIL13 and DMSO/stattic (n=3). ***p < 0.001, **p < 0.01.

We next examined the molecular mechanisms by which IL13 promotes gastric cancer stemness. STAT3 is a major regulatory factor in maintaining the stemness of tumor cells (23). We observed that the phosphorylation of STAT3 (p-STAT3) was increased in GC cells treated with the IL13 recombinant protein (**Figure 7A**; **Supplementary Figure 4A**). Therefore, we assume that the promotion effects on GC cells by IL13 is STAT3 dependent. To target STAT3 signaling, we used Stattic (STAT3 inhibitor). Stattic abrogated the self-renewal, migration and colony formation of gastric cancer cells induced by IL-13 (**Figures 7B–G**). Consistently, Stattic also abrogated the expression of the stemness-related markers *sox2*, *lgr5* and *nanog* induced by IL-13 (**Figure 7H**; **Supplementary Figure 4B**). These results indicate that STAT3 activation is necessary for IL-13-promoted cancer cell stemness.

## 3 Materials and methods

### 3.1 Patients

Tissue was obtained from the Affiliated Hospital of Jining Medical University. The cancer tissue samples were obtained from GC patients with untreated, primary, nonmetastatic gastric tumors who underwent GC resection. Adjacent normal gastric tissue was collected more than 5 cm from the carcinoma tissue. The patients gave written consent. The studies involving human participants were reviewed and approved by the Ethics Committee of the Affiliated Hospital of Jining Medical College, ethical approval number 2022B034.

### 3.2 Single-cell sequencing

After surgical excision, the tumor tissues were processed to prepare single-cell suspensions. The tissue samples were cut into small pieces, and the cells were washed twice with precooled RPMI 1640 + 0.04% BSA medium under aseptic conditions. The tissues were cut into small pieces of approximately 0.5 mm^3^ using surgical scissors, placed in freshly prepared hydrolase and digested at a constant temperature of 37°C for 60 minutes, mixing every 10 minutes. The tissue was filtered 2 times using a cell sieve and centrifuged at 300 × g for 5 minutes at 4°C. After resuspending the sediment with an appropriate amount of medium, an equal volume of erythrocyte lysis buffer (MACS, catalog number: 130-094-183) was added. After mixing, the cells were incubated at 4°C for 10 minutes and centrifuged at 300 x g for 5 minutes, and the Dead Cell Removal Kit was used to ensure that the cell viability rate was greater than 90%. The 10x Genomics platform, which utilizes microfluidics to encase cells and beads with a cell barcode in a droplet, was employed. These droplets were collected, and the cells within them were lysed, allowing for mRNA within the cells to attach to the cell barcode on the bead. This process results in the formation of single-cell GEMs. These GEMs are then reverse transcribed to create a cDNA library within the droplet. The sample source for the target sequence is distinguished by the sample index on the library sequence. Quality control for raw offline data was evaluated using FastQC software, while data quality was assessed by counting raw data using CellRanger and comparing it to the reference genome. The Seurat software suite was used for additional quality assurance and data processing. The high-quality cells in each sample were quantified as follows. After excluding cell doublets, cell clusters, and apoptotic cells for quality control, the final number of cells obtained ranged from 6156 to 10412; the average UMI number in each cell ranged from 4283 to 5616; and the average genes in each cell ranged from 1373 to?. Between 0.0584 and 0.0681 mitochondrial genes per cells were found on average.

### 3.3 Cell culture

AGS and HGC-27 (Chinese Academy of Sciences Cell Bank) cells were grown in DF-12/DMEM supplemented with 10% fetal bovine serum (FBS, Gibco) at 37°C with 5% CO2.

### 3.4 Treg isolation

Tissues were handled into single-cell suspensions and incubated with antibodies against CD45, CD3, CD25, and TNF, and enriched cells were sorted using a BD FACS Aria II cell sorter (Becton Dickinson, Franklin Lakes, NJ) and then cultured with Treg Expansion Kit (Miltenyi) reagents, IL-2 (Thermo Fisher), and TexMACS™ GMP Medium (Alfa Aesar).

### 3.5 Pathway and functional annotation analysis

The differentially expressed genes (DEGs) were identified by the service provider (oebiotech). GO enrichment and KEGG enrichment analysis of the DEGs was performed *via* www.xiantao.love.

### 3.6 Differential gene expression and prognostic performance of the DEGs in TCGA samples

For TCGA STAD project level 3 HTSeq-FPKM format RNAseq data, the fragments per kilobase per million mapped reads (FPKM)-formatted RNAseq data were converted into transcripts per million mapped reads (TPM) format and log2-converted, and paired samples were retained. A Kaplan–Meier curve was constructed to compare the overall survival rate (http://kmplot.com/analysis/index.php?p=service &cancer=gastric).

### 3.7 Multiple fluorescence staining

Multiplex fluorescence according to the manufacturer’s instructions (Absin, abs50012).

### 3.8 Culture of spheroid cells

A total of 5000 cells were inoculated in low-adhesion tissue culture plates with serum-free medium and 20 ng/mL EGF, 10 ng/mL bFGF, and 2% B27.

### 3.9 RNA extraction and quantitative real-time PCR

The cells were fully lysed with TRIzol, and then trichloromethane, isopropanol and alcohol were added to extract RNA. According to the manufacturer’s instructions, the RNA was reverse transcribed into cDNA, and then qPCR was performed (HiScript III RT SuperMix, Vazyme Biotech Co., Ltd; SYBR Green PCR kit, Toyobo). The primers used for qRT−PCR are listed in **Table S1**.

### 3.10 WB

Cell protein was extracted by lysis (Beyotime) and protease inhibitor (Boster Biological Technology). Equal amounts of total protein were separated on 10% SDS-polyacrylamide gels and transferred to nitrocellulose membranes (Millipore). The primary antibody was incubated with the membranes overnight at 4°C (**Table S2**), and the secondary antibody (**Table S2**) was incubated with the membranes for 1 hour at room temperature. The protein bands were detected according to the manufacturer’s instructions (Vazyme).

### 3.11 Cell growth assay

A total of 1000 cells were seeded in 6-well plates. After 14 days, the cells were washed with PBS and stained with 1% crystal violet. In addition, 2000 cells were plated in 96-well plates, and CCK-8 solution was added at different times to detect the OD value.

### 3.12 Flow cytometry analysis

Tissues or adherent cells were processed into single-cell suspensions and stained using the antibodies listed in **Table S1**. The cells were analyzed by flow cytometry in a CytoFLEX Flow Cytometer. The data were analyzed using FlowJo software. The appropriate isotype controls were used. Control antibodies were used for gating.

### 3.13 Cell migration

A total of 10^5^ cells were seeded on Transwell plates, and the lower chamber was filled with DMEM supplemented with 15% FBS. The cells were allowed to migrate for 18 hours. The cells that migrated to the lower surface of the filter were fixed with 4% paraformaldehyde and stained with 1% crystal violet. The stained cells were observed under a microscope.

### 3.14 *In vivo* tumor xenograft models

Cells were injected subcutaneously into NVSG mice (4-6 weeks, IL-2 knockout), which were obtained from Beijing Viewsolid Biotech Co. Ltd. The mice were anesthetized and sacrificed when they had lost approximately 20% of their body weight and when the tumor diameter reached a maximum of approximately 2 cm. The xenograft tumors of each group were removed, weighed, and fixed in formalin. The animal studies were reviewed and approved by the Ethics Committee of the Affiliated Hospital of Jining Medical College (ethical approval number 2021-11-B008).

### 3.15 Statistical analyses

The calculation of differentially expressed genes in Treg was performed using a two-sided Wilcoxon test (24). Statistical analyses were performed using GraphPad Prism 7 and ImageJ software. Two-tailed and unpaired Student’s t tests were used for comparison of two groups. One-way ANOVA tests were used for multiple group comparisons. Survival curves were estimated using the Kaplan−Meier method and compared using the log-rank test. Data are shown as the mean ± SD. p<0.05 was considered to indicate statistical significance.

## 4 Discussion

Gastric cancer is one of the most common malignancies, and the underlying mechanism of gastric cancer progression is still unclear. Traditional treatment methods have certain limitations. Single-cell sequencing enables expression profiling of individual cells within entire tissues or tumors (25). In this study, we analyzed a cell atlas of GC tissues and GM tissues and found increased infiltration of Tregs in GC tissues. Tregs are important components of the tumor microenvironment (TME), and Tregs utilize numerous mechanisms to promote tumor evasion from the immune system. Previous studies have proven that the increase in the number of Tregs in the GC microenvironment has prognostic significance and a key role in promoting the progression of GC (26). However, the mechanism underlying the regulation of the occurrence and development of gastric cancer by Tregs is not well elucidated.

By analyzing the upregulated genes of Tregs in tumors, TNF was identified as the hub gene. To confirm the reliability of the hub genes, we verified the high expression of TNF in tumor Tregs and found that the high expression of TNF was significantly related to the prognosis of gastric cancer patients. In our study, we found a new TNF+ Treg subset. Previous studies have shown that TNF is involved in the malignant progression of tumors; for example, before tumor cell inoculation, stimulating tumor cells with recombinant TNF and transducing tumor cells with TNF enhanced the metastatic potential of tumor cells *in vivo (* 27, 28). Commonly, TNF activates Tregs through TNFR2 or IL2 signaling, leading to inflammatory reactions in autoimmunity, sepsis, infection and tumors (29, 30). Here, we speculated that the newly identified TNF+ Tregs might be a subpopulation that can autocrine TNF and proved that TNF+ Tregs participate in the malignant development of gastric cancer. Our study indicates that TNF+ Tregs in GCs significantly promote the proliferation, migration and colony formation of gastric cancer cells. In normal tissues, TNF+ Tregs account for approximately 1% of Tregs, whereas in tumor tissues, TNF+ Tregs account for approximately 33% of Tregs. The expression of TNF in Treg cells appears to control not only their function but also their number.

This study provides several pieces of evidence to demonstrate that TNF+ Tregs can directly promote the stemness of gastric cancer cells: TNF+ Tregs can (1) promote the sphere formation of gastric cancer cells and (2) enhance the tumorigenicity of gastric cancer cells. Tregs in the tumor microenvironment have been shown to regulate tumor cell stemness through IL10 (12), TGF-β (14), etc. Moreover, recent studies have revealed that high levels of TNF can promote the stemness of breast cancer and kidney cancer cells (19, 20). Our study shows that TNF+ Tregs affect the stemness of gastric cancer cells through IL-13 secretion. IL-13 is an important mediator of immunosuppression, and previous studies have suggested that IL-13 can be secreted by Tregs (31). IL13 contributes to the oncogenic effects of renal cell carcinoma and chronic lymphocytic leukemia (32). In addition, IL-13 promotes the biosynthesis of cytidine deaminase in colonic epithelial cells, conferring the potential to activate the inflammatory-cancer transition in the intestine (21). Until now, there has been no evidence suggesting that IL-13 can directly enhance the stemness of gastric cancer cells. In this study, IL13 significantly promoted the self-renewal of gastric cancer cells and increased the expression of *sox2*, *lgr5* and *prom1*. STAT3 is involved in maintaining the expression of genes encoding stem cell phenotypes; therefore, it is used as a stemness marker (23). Our study shows that inhibition of STAT3 phosphorylation significantly reduces the regulatory effect of IL13 on cancer cell stemness.

In this paper, we investigated the promotion effects of TNF+ Tregs on stemness in gastric cancer cells through the IL13/STAT3 signaling pathway. In the future, the crosstalk between gastric cancer cells and the surface proteins expressed by Tregs should be investigated. This study may provide more evidence and therapeutic strategies to disrupt the interaction between Tregs and gastric cancer cells, thus improving the prognosis of gastric cancer patients.

## Data availability statement

The datasets presented in this study can be found in online repositories. The names of the repository/repositories and accession number(s) can be found in the article/**Supplementary Material**.

## Author contributions

QB, RZ, and ZH conceived the original idea. RZ developed the necessary methodology and designed and performed *in vitro* and *in vivo* experiments. BaZ and LW performed *in vivo* assays, reviewed the manuscript, and provided critical suggestions during the project execution. XZ and BH reviewed the manuscript and provided critical support during the experimental design. MJ assisted in the collection of gastric cancer tissue. All authors discussed the results and commented on the manuscript. All authors contributed to the article and approved the submitted version.

## References

[1] Sun J Cao Q Lin S Chen Y Liu X Hong Q. Knockdown of CALM2 increases the sensitivity to afatinib in HER2-amplified gastric cancer cells by regulating the Akt/FoxO3a/Puma axis. Toxicol Vitro: Int J Published Assoc BIBRA (2023) 87:105531. doi: 10.1016/j.tiv.2022.105531 36460225

[2] Alsadat Mahmoudian R Amirhosein M Mahmoudian P Fardi Golyan F Mokhlessi L Maftooh M. The therapeutic potential value of cancer-testis antigens in immunotherapy of gastric cancer. Gene (2023) 853:147082. doi: 10.1016/j.gene.2022.147082 36464170

[3] Yang T Shu X Zhang HW Sun LX Yu L Liu J. Enolase 1 regulates stem cell-like properties in gastric cancer cells by stimulating glycolysis. Cell Death Dis (2020) 11:870. doi: 10.1038/s41419-020-03087-4 33067426 PMC7567818

[4] Bergerhoff K Pedersen M. Isolation and analysis of tumor-infiltrating treg. Methods Mol Biol (Clifton NJ) (2023) 2559:51–63. doi: 10.1007/978-1-0716-2647-4_5 36180626

[5] Wang J Gong R Zhao C Lei K Sun X Ren H. Human FOXP3 and tumour microenvironment. Immunology (2023) 168:248–55. doi: 10.1111/imm.13520 35689826

[6] Lim TY Perpiñán E Londoño MC Miquel R Ruiz P Kurt AS. Low dose interleukin-2 selectively expands circulating regulatory T cells but fails to promote liver allograft tolerance in humans. J Hepatol (2023) 78:153–64. doi: 10.1016/j.jhep.2022.08.035 36087863

[7] Wu Z Zhang C Najafi M. Targeting of the tumor immune microenvironment by metformin. J Cell Commun Signaling (2022) 16:333–48. doi: 10.1007/s12079-021-00648-w PMC9411277 34611852

[8] Bayik D Lathia JD. Cancer stem cell-immune cell crosstalk in tumour progression. Nat Rev Cancer (2021) 21:526–36. doi: 10.1038/s41568-021-00366-w PMC8740903 34103704

[9] Jinushi M Chiba S Yoshiyama H Masutomi K Kinoshita I Dosaka-Akita H. Tumor-associated macrophages regulate tumorigenicity and anticancer drug responses of cancer stem/initiating cells. Proc Natl Acad Sci United States America (2011) 108:12425–30. doi: 10.1073/pnas.1106645108 PMC3145680 21746895

[10] Panni RZ Sanford DE Belt BA Mitchem JB Worley LA Goetz BD. Tumor-induced STAT3 activation in monocytic myeloid-derived suppressor cells enhances stemness and mesenchymal properties in human pancreatic cancer. Cancer Immunol Immunother: CII (2014) 63:513–28. doi: 10.1007/s00262-014-1527-x PMC3994288 24652403

[11] Wang F Xia H Yao S. Regulatory T cells are a double-edged sword in pulmonary fibrosis. Int Immunopharmacol (2020) 84:106443. doi: 10.1016/j.intimp.2020.106443 32334385

[12] Xu Y Mou J Wang Y Zhou W Rao Q Xing H. Regulatory T cells promote the stemness of leukemia stem cells through IL10 cytokine-related signaling pathway. Leukemia (2022) 36:403–15. doi: 10.1038/s41375-021-01375-2 34381181

[13] Xu Y Dong X Qi P Ye Y Shen W Leng L. Sox2 communicates with tregs through CCL1 to promote the stemness property of breast cancer cells. Stem Cells (Dayton Ohio) (2017) 35:2351–65. doi: 10.1002/stem.2720 PMC5958902 29044882

[14] Liu S Zhang C Wang B Zhang H Qin G Li C. Regulatory T cells promote glioma cell stemness through TGF-β-NF-κB-IL6-STAT3 signaling. Cancer Immunol Immunother: CII (2021) 70:2601–16. doi: 10.1007/s00262-021-02872-0 PMC8360896 33576874

[15] Taz TA Ahmed K Paul BK Kawsar M Aktar N Mahmud SMH. Network-based identification genetic effect of SARS-CoV-2 infections to idiopathic pulmonary fibrosis (IPF) patients. Briefings Bioinf (2021) 22:1254–66. doi: 10.1093/bib/bbaa235 PMC7665362 33024988

[16] Pang E Hao Y Sun Y Lin K. Differential variation patterns between hubs and bottlenecks in human protein-protein interaction networks. BMC Evol Biol (2016) 16:260. doi: 10.1186/s12862-016-0840-8 27903259 PMC5131443

[17] Keren L Bosse M Thompson S Risom T Vijayaragavan K McCaffrey E. MIBI-TOF: a multiplexed imaging platform relates cellular phenotypes and tissue structure. Sci Adv (2019) 5:eaax5851. doi: 10.1126/sciadv.aax5851 31633026 PMC6785247

[18] Chen Y Wen H Zhou C Su Q Lin Y Xie Y. TNF-α derived from M2 tumor-associated macrophages promotes epithelial-mesenchymal transition and cancer stemness through the wnt/β-catenin pathway in SMMC-7721 hepatocellular carcinoma cells. Exp Cell Res (2019) 378:41–50. doi: 10.1016/j.yexcr.2019.03.005 30844387

[19] Liu W Lu X Shi P Yang G Zhou Z Li W. TNF-α increases breast cancer stem-like cells through up-regulating TAZ expression *via* the non-canonical NF-κB pathway. Sci Rep (2020) 10:1804. doi: 10.1038/s41598-020-58642-y 32019974 PMC7000832

[20] Zhang L Jiao M Wu K Li L Zhu G Wang X. TNF-α induced epithelial mesenchymal transition increases stemness properties in renal cell carcinoma cells. Int J Clin Exp Med (2014) 7:4951–8. PMC4307439 25663992

[21] He B Liang J Zhang Y Shi S Cao J Zhang Z. IL-13/IL-13RA2 signaling promotes colorectal cancer stem cell tumorigenesis by inducing ubiquitinated degradation of p53. Genes Dis Inpress (2023). doi: 10.1016/j.gendis.2023.01.027 PMC10425805 37588218

[22] Li X Liu M Shi Q Fang Y Fu D Shen ZX. Elevated serum IL-13 level is associated with increased treg cells in tumor microenvironment and disease progression of diffuse large b-cell lymphoma. Hematol Oncol (2022) 41(2):230–8. doi: 10.1002/hon.2993 35304777

[23] Wei S Li J Tang M Zhang K Gao X Fang L. STAT3 and p63 in the regulation of cancer stemness. Front Genet (2022) 13:909251. doi: 10.3389/fgene.2022.909251 36061200 PMC9428145

[24] Schlesinger Y Yosefov-Levi O Kolodkin-Gal D Granit RZ Peters L Kalifa R. Single-cell transcriptomes of pancreatic preinvasive lesions and cancer reveal acinar metaplastic cells’ heterogeneity. Nat Commun (2020) 11:4516. doi: 10.1038/s41467-020-18207-z 32908137 PMC7481797

[25] Wu F Fan J He Y Xiong A Yu J Li Y. Single-cell profiling of tumor heterogeneity and the microenvironment in advanced non-small cell lung cancer. Nat Commun (2021) 12:2540. doi: 10.1038/s41467-021-22801-0 33953163 PMC8100173

[26] Liu X Zhang Z Zhao G. Recent advances in the study of regulatory T cells in gastric cancer. Int Immunopharmacol (2019) 73:560–7. doi: 10.1016/j.intimp.2019.05.009 31181438

[27] Yang A Fan H Zhao Y Chen X Zhu Z Zha X. An immune-stimulating proteoglycan from the medicinal mushroom huaier up-regulates NF-κB and MAPK signaling *via* toll-like receptor 4. J Biol Chem (2019) 294:2628–41. doi: 10.1074/jbc.RA118.005477 PMC6393594 30602571

[28] Mandal RK Khan MA Hussain A Akhter N Jawed A Dar SA. A trial sequential meta-analysis of TNF-α -308G>A (rs800629) gene polymorphism and susceptibility to colorectal cancer. Biosc Rep (2019) 39(1):BSR20181052. doi: 10.1042/bsr20181052 PMC6331670 30509964

[29] Chen X Oppenheim JJ. TNF-alpha: an activator of CD4+FoxP3+TNFR2+ regulatory T cells. Curr Dir Autoimmun (2010) 11:119–34. doi: 10.1159/000289201 PMC6314650 20173391

[30] Ronin E Pouchy C Khosravi M Hilaire M Grégoire S Casrouge A. Tissue-restricted control of established central nervous system autoimmunity by TNF receptor 2-expressing treg cells. Proc Natl Acad Sci USA (2021) 118(13):e2014043118. doi: 10.1073/pnas.2014043118 33766913 PMC8020675

[31] Liu Q Dwyer GK Zhao Y Li H Mathews LR Chakka AB. IL-33-mediated IL-13 secretion by ST2+ tregs controls inflammation after lung injury. JCI Insight (2019) 4(6):e123919. doi: 10.1172/jci.insight.123919 30779711 PMC6482994

[32] Dalessandri T Crawford G Hayes M Castro Seoane R Strid J. IL-13 from intraepithelial lymphocytes regulates tissue homeostasis and protects against carcinogenesis in the skin. Nat Commun (2016) 7:12080. doi: 10.1038/ncomms12080 27357235 PMC4931319

