# Supplementary material for: TNF+ regulatory T cells regulate the stemness of gastric cancer cells through the IL13/STAT3 pathway
Source: Front Oncol. 2023 Jul 18;13:1162938. doi: 10.3389/fonc.2023.1162938 (PMC10392945; doi:10.3389/fonc.2023.1162938)
Supplement: Supplementary file 1 [file DataSheet_1.docx]

Raw data：https://www.jianguoyun.com/c/sd/16c9450/10215aca034e9a84
