# Supplementary material for: TNF+ regulatory T cells regulate the stemness of gastric cancer cells through the IL13/STAT3 pathway
Source: Front Oncol. 2023 Jul 18;13:1162938. doi: 10.3389/fonc.2023.1162938 (PMC10392945; doi:10.3389/fonc.2023.1162938)
Supplement: Supplementary file 2 [file DataSheet_2.docx]

Supplementary Material

TNF+ Tregs regulate the stemness of gastric cancer cells through the IL13/STAT3 pathway

Rou Zhao, Zhun He*, Qingli Bie*

*** Correspondence:** Corresponding Author: Qingli Bie xiaobie890101@163.com

Zhun He hezhun69@163.com

## Supplementary Figures

**Figure S1.** Violin plot of marker gene expression in cell clusters.

**Figure S2.** (A) A heatmap based on normalized expression was used to show the top eighty differentially expressed genes of Tregs in GC tissue and adjacent normal tissue. (B)Flow sorting of TNF+ Tregs and TNF-Tregs from gastric cancer, detection of differential expression of hub genes in TNF+ Tregs and TNF-Tregs in Figure 2A. (C) Expression differences were calculated by selecting 27 pairs of gastric cancer and adjacent normal tissues. (D)The relationships between TNF and Treg infiltration levels in gastric cancer. (E)Correlation diagrams showing the differences in Treg infiltration levels between the TNF-high and TNF-low groups.

**Figure S3.** Determination of IL13 in supernatants of TNF+/TNF-Tregs in gastric cancer tissues and TNF+Tregs in adjacent normal tissues.

**Figure S4.** (A)The quantification of figure7A Western Blotting. (B) Detection of SOX2, LGR5 and CD133 protein expression.
