# Supplementary figures and images for: TNF+ regulatory T cells regulate the stemness of gastric cancer cells through the IL13/STAT3 pathway
Source: Front Oncol. 2023 Jul 18;13:1162938. doi: 10.3389/fonc.2023.1162938 (PMC10392945; doi:10.3389/fonc.2023.1162938)

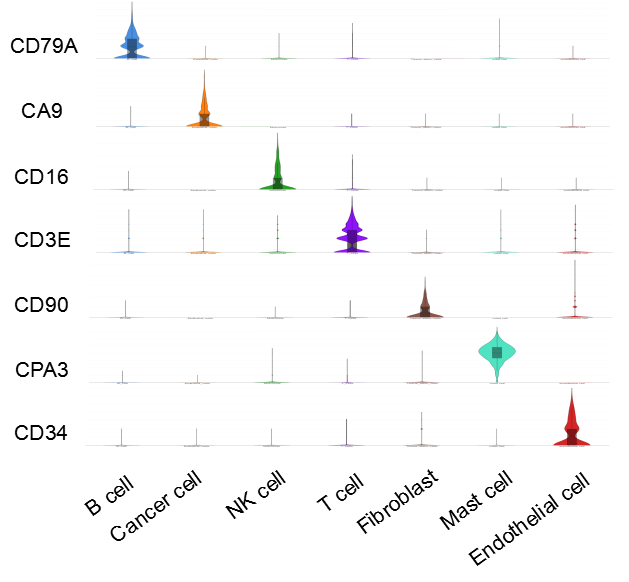

Supplement: Supplementary file 3 [file Image_1.tif]

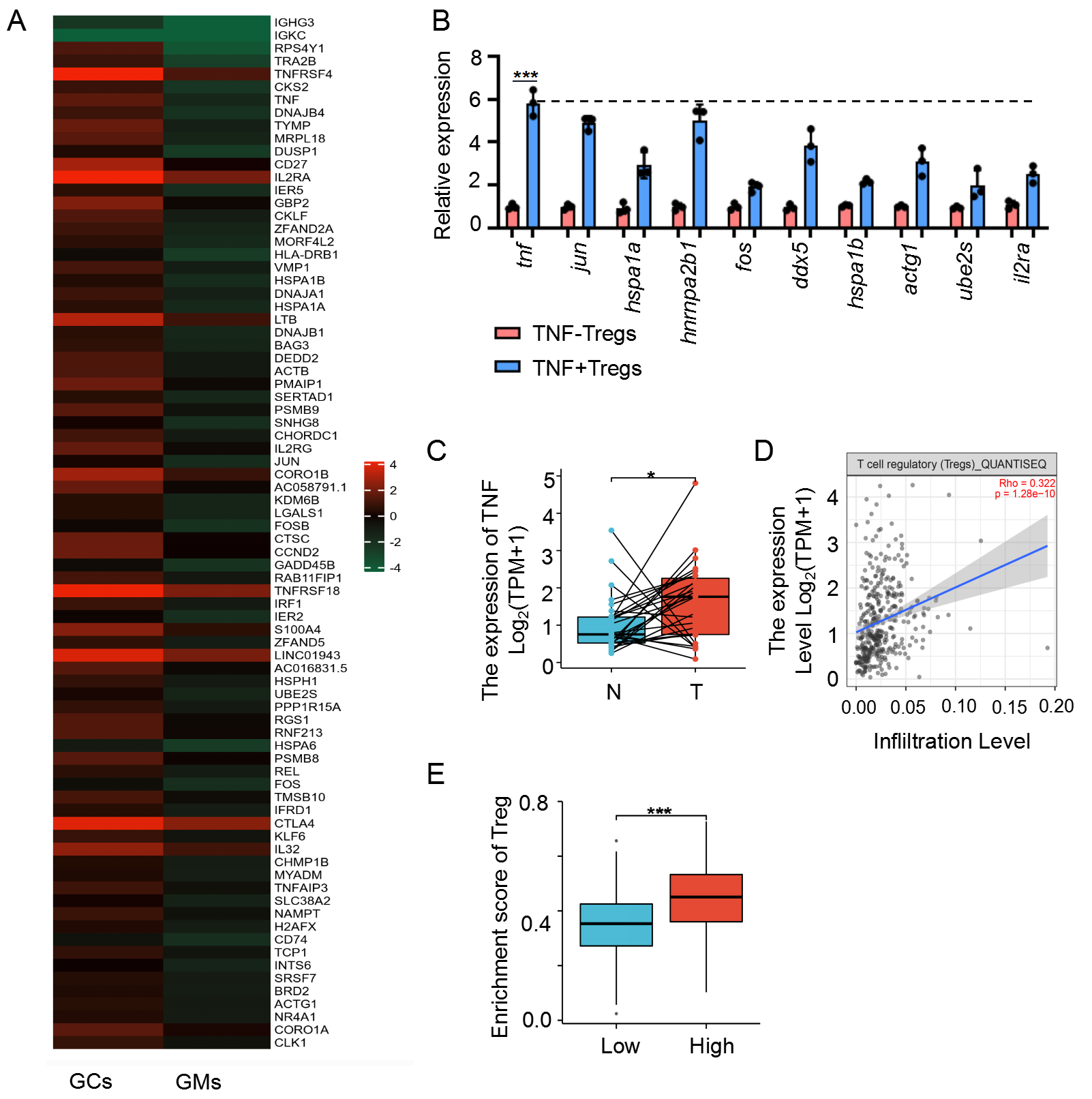

Supplement: Supplementary file 4 [file Image_2.tif]

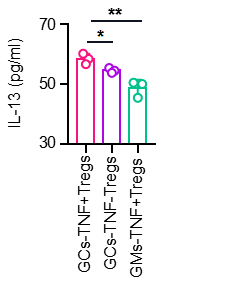

Supplement: Supplementary file 5 [file Image_3.tif]

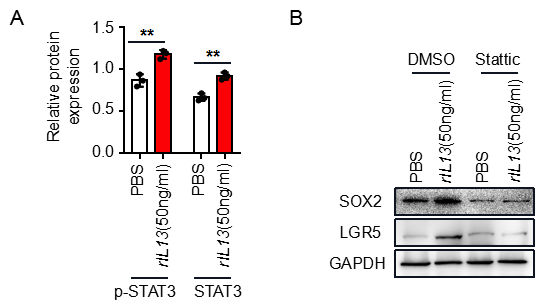

Supplement: Supplementary file 6 [file Image_4.tif]
