# Supplementary material for: TNF+ regulatory T cells regulate the stemness of gastric cancer cells through the IL13/STAT3 pathway
Source: Front Oncol. 2023 Jul 18;13:1162938. doi: 10.3389/fonc.2023.1162938 (PMC10392945; doi:10.3389/fonc.2023.1162938)
Supplement: Supplementary file 7 [file Table_1.docx]

Supplementary Table 1. Sequences of real-time PCR primers

| mRNA | Primer | Sequences (5′-3 ′) | Annealing temperature |
| --- | --- | --- | --- |
| Human-*cd133* | Forward | GAGCTAAGGGAAGGGCGG | 60°C |
|  | Reverse | TTCTGTCTGAGGCTGGCTTG |  |
| Human-*nanog* | Forward | CCTGATTCTTCCACCAGTCC | 60°C |
|  | Reverse | TGCTATTCTTCGGCCAGTTG |  |
| Human-*lgr5* | Forward | CATCAGCTATGTGCCCCCAA | 62°C |
|  | Reverse | TGTGGAGCCCATCAAAGCAT |  |
| Human-*β-actin* | Forward | GACCTGTACGCCAACACAGT | 59°C |
|  | Reverse | CTCAGGAGGAGCAATGATCT |  |
| Human-*sox2* | Forward | ACACCAATCCCATCCACACT | 60°C |
|  | Reverse | GCAAACTTCCTGCAAAGCTC |  |
| Human-*tnf* | Forward | CCCATGTTGTAGCAAACCCT | 60°C |
|  | Reverse | ATGAGGTACAGGCCCTCTGA |  |
| Human-*actg1* | Forward | CCGAGCCGTGTTTCCTTCC | 62°C |
|  | Reverse | GCCATGCTCAATGGGGTACT |  |
| Human-*jun* | Forward | TCCAAGTGCCGAAAAAGGAAG | 60°C |
|  | Reverse | CGAGTTCTGAGCTTTCAAGGT |  |
| Human-*hspa1a* | Forward | AGCTGGAGCAGGTGTGTAAC |  |
|  | Reverse | CAGCAATCTTGGAAAGGCCC |  |
| Human-*hspa1b* | Forward | TTTGAGGGCATCGACTTCTACA | 61°C |
|  | Reverse | CCAGGACCAGGTCGTGAATC |  |
| Human-*fos* | Forward | CCGGGGATAGCCTCTCTTACT | 62°C |
|  | Reverse | CCAGGTCCGTGCAGAAGTC |  |
| Human-*il2ra* | Forward | GTGGGGACTGCTCACGTTC | 62°C |
|  | Reverse | CCCGCTTTTTATTCTGCGGAA |  |
| Human-*ddx5* | Forward | ATGTCGGGTTATTCGAGTGACC | 62°C |
|  | Reverse | TGTGCGCCTAGCCAAATCAG |  |
| Human-*hnrnpa2b1* | Forward | ATTGATGGGAGAGTAGTTGAGCC | 61°C |
|  | Reverse | AATTCCGCCAACAAACAGCTT |  |
| Human-*ube2s* | Forward | ACAAGGAGGTGACGACACTGA | 61°C |
|  | Reverse | CCACGTTCGGGTGGAAGAT |  |
