# Supplementary material for: TNF+ regulatory T cells regulate the stemness of gastric cancer cells through the IL13/STAT3 pathway
Source: Front Oncol. 2023 Jul 18;13:1162938. doi: 10.3389/fonc.2023.1162938 (PMC10392945; doi:10.3389/fonc.2023.1162938)
Supplement: Supplementary file 8 [file Table_2.docx]

Supplementary Table 2. Antibodies

| Primary Antibody | Source | Detection and dilution ratio | Manufacturer |
| --- | --- | --- | --- |
| GAPDH | Rabbit | WB1:10000 | Bioworld |
| STAT3 | Mice | WB1:1000 | Cell signaling technology |
| pSTAT3 | Rabbit | WB1:1000 | Cell signaling technology |
| LGR5 | Rabbit | WB1:1000 | Abclonal |
| Sox2 | Mice | WB1:1000 | Cell signaling technology |
| Anti-Rabbit antibody-HRP |  | WB1:2000 | Cell signaling technology |
| Anti-Mice antibody-HRP |  | WB1:2000 | Cell signaling technology |
| Anti-IL13 |  |  | Cendakimab |
| PE anti-human CD45 Antibody |  | Fc 1:200 | BioLegend |
| FITC anti-human CD25 Antibody |  | Fc 1:200 | BioLegend |
| APC anti-human CD3 Antibody |  | Fc 1:200 | BioLegend |
| APC anti-human FOXP3 Antibody |  | Fc 1:200 | BioLegend |
| TNF |  | Fc 1:200 | Abcam |
| IL13 |  | Fc 1:200 | Invitrogen |
